# Percutaneous coronary intervention outcomes in patients with stable coronary disease and left ventricular systolic dysfunction

**DOI:** 10.1002/ehf2.12510

**Published:** 2019-09-27

**Authors:** Adam D. DeVore, Eric Yow, Mitchell W. Krucoff, Matthew W. Sherwood, Linda K. Shaw, Karen Chiswell, Christopher M. O'Connor, Erik Magnus Ohman, Eric J. Velazquez

**Affiliations:** ^1^ Department of Medicine Duke University School of Medicine Durham NC USA; ^2^ Duke Clinical Research Institute Duke University School of Medicine 200 Morris Street, 6318 Durham NC 27701 USA; ^3^ Inova Heart and Vascular Institute Falls Church VA USA; ^4^ Yale University School of Medicine New Haven CT USA

**Keywords:** Coronary artery disease, Left ventricular dysfunction, Heart failure, Outcomes

## Abstract

**Aims:**

We sought to better understand the role of percutaneous coronary intervention (PCI) in patients with stable coronary artery disease (CAD) and moderate or severe left ventricular systolic dysfunction.

**Methods and results:**

Using data from the Duke Databank for Cardiovascular Disease, we analysed patients who underwent coronary angiography at Duke University Medical Center (1995–2012) that had stable CAD amenable to PCI and left ventricular ejection fraction ≤35%. Patients with acute coronary syndrome or Canadian Cardiovascular Society class III or IV angina were excluded. We used propensity‐matched Cox proportional hazards to evaluate the association of PCI with mortality and hospitalizations. Of 901 patients, 259 were treated with PCI and 642 with medical therapy. PCI propensity scores created from 24 variables were used to assemble a matched cohort of 444 patients (222 pairs) receiving PCI or medical therapy alone. Over a median follow‐up of 7 years, 128 (58%) PCI and 125 (56%) medical therapy alone patients died [hazard ratio 0.87 (95% confidence interval 0.68, 1.10)]; there was also no difference in the rate of a composite endpoint of all‐cause mortality or cardiovascular hospitalization [hazard ratio 1.18 (95% confidence interval 0.96, 1.44)] between the two groups.

**Conclusions:**

In this well‐profiled, propensity‐matched cohort of patients with stable CAD amenable to PCI and moderate or severe left ventricular systolic dysfunction, the addition of PCI to medical therapy did not improve long‐term mortality, or the composite of mortality or cardiovascular hospitalization. The impact of PCI on other outcomes in these high‐risk patients requires further study.

## Introduction

Heart failure (HF) affects more than 5 million adults in the United States alone, and the prevalence is expected to increase.^1^ Despite recent advances in the treatment of chronic HF,^2^, ^3^ patient outcomes remain poor, and there is an unmet need to identify and treat other targets. Reversible ischaemia and infarction related to coronary artery disease (CAD) remains one of the most common aetiologies of HF,^4^, ^5^ yet the role of non‐surgical revascularization in addition to medical therapy in the management of HF remains unclear.

The Surgical Treatment for Ischemic Heart Failure (STICH) trial compared medical therapy alone with medical therapy plus coronary artery bypass grafting (CABG) in 1212 patients with CAD and left ventricular systolic dysfunction (LVSD).^6^ Enrolled patients had a left ventricular ejection fraction (LVEF) ≤35% and CAD amenable to CABG. Patients were excluded if they had a recent acute myocardial infarction or Canadian Cardiovascular Society (CCS) class III or IV angina. The results of STICH, combined with long‐term follow‐up from the STICH Extension Study (STICHES),^7^ provided randomized clinical trial evidence that CABG in addition to medical therapy improves all‐cause mortality and cardiovascular hospitalizations for patients with LVSD. There is operative risk with surgical revascularization that may be mitigated by percutaneous coronary intervention (PCI); however, the most recent PCI guidelines^8^ noted insufficient data to make a recommendation for or against PCI as a treatment to improve survival in patients with significant CAD and LVSD.

We used data from the Duke Databank for Cardiovascular Disease to explore the impact of PCI in addition to medical therapy on outcomes for patients with significant CAD and moderate or severe LVSD. We studied a population of patients similar to those in STICH and hypothesized that patients treated with PCI in addition to medical therapy would have improved survival compared with those treated with medical therapy alone, as well as lower rates of a composite endpoint of death or cardiovascular hospitalization.

## Methods

### Data sources

The Duke Databank for Cardiovascular Disease is a databank of all patients undergoing cardiac catheterization and/or cardiac surgery at Duke University Medical Center since 1969. Data from the index catheterization are prospectively collected as part of routine patient care. Baseline clinical variables for each patient are collected and stored in the databank using methods previously described.^9^ Follow‐up for these patients is acquired through self‐administered questionnaires, with telephone follow‐up to non‐responders. Annual surveys collect data on overall health, hospitalizations, myocardial infarction, stroke, cardiac procedures, and medication use. Patients are surveyed 6 months after the index visit and yearly thereafter. Patients not contacted through this mechanism have vital status determined through a search of the National Death Index.^10^

The Duke Echocardiography Laboratory Database is a prospectively maintained digital archive of all clinical echocardiograms performed at Duke University Medical Center since 1995 that is linked to a corresponding searchable reporting database. We also utilized information from Duke University Medical Center administrative and clinical databases for variables not available in the Duke Databank for Cardiovascular Disease or Duke Echocardiography Laboratory Database, such as medication use. The institutional review board of the Duke University Health System approved this study and provided a waiver for informed consent in the compilation and reporting of the data.

### Study population and definitions

Patients were included in this analysis if they had (i) coronary angiography from 1 January 1995 to 31 December 2012, (ii) CAD potentially amenable to PCI, and (iii) LVEF ≤35%. CAD potentially amenable to PCI was defined by significant stenosis in ≥1 epicardial coronary vessel, although angiograms were not reviewed to determine the level of risk associated with a PCI (e.g. complexity of coronary anatomy). The definition of significant stenosis changed during the study period. Prior to 1 July 2007, stenosis was considered significant if ≥75%. After this date, stenosis was considered significant if ≥50%. Stenosis in the left main was handled the same as stenosis in other vessels. The LVEF was determined by either ventriculography or echocardiography. In the event that patients had both studies performed, then data from the ventriculogram were used because the ventriculogram was performed at the time of the coronary angiogram.

For purposes of this study, we excluded patients for any of the following reasons: a history of prior CABG surgery; unknown LVEF; recent acute coronary syndrome (≤30 days prior to angiogram); CCS class III or IV angina per the patient history obtained just before coronary angiogram by a treating health care provider; valve disease or congenital heart disease as the primary indication for cardiac catheterization; significant co‐morbid conditions likely to affect survival including advanced kidney disease requiring dialysis, history of malignancy, or severe liver disease; and those who had CABG ≤30 days after the angiogram, because CABG was interpreted as the intended treatment strategy.

Treatment strategies were determined ≤30 days after the coronary angiogram. If a patient received a PCI ≤30 days after the coronary angiogram, then he or she was considered to be treated with PCI in addition to medical therapy and follow‐up began after the PCI. In order to ensure that a patient was not intended for CABG, a patient with no PCI or CABG ≤30 days of the angiogram was considered to be treated with medical therapy alone and follow‐up began 5 days after the angiogram (because 5 days was the mean time between cardiac catheterization and CABG at our institution, and to be consistent with a prior study^11^). Other early deaths in the medical arm were examined to make sure the patients were not waiting for a scheduled CABG or PCI. Medication use was defined by prescribed medications noted within 30 days before or after the coronary angiogram.

### Statistical methods

We summarized patient characteristics for the full study population by reporting medians with interquartile ranges for continuous variables and frequencies with percentages for categorical variables. Comparisons for continuous variables were made using Kruskal–Wallis tests, and categorical variables were compared using Chi‐square tests.

A multivariable logistic regression model predicting the propensity to receive PCI compared with medical therapy was constructed. Investigators reviewed and selected variables for the propensity model based on clinical judgment and included 24 different variables (including age, LVEF, co‐morbid conditions, and medications). The full list of variables is listed in the Supporting Information, *Figure* *S1*. Variables with missing >15% were not selected for the model. Variables with missing ≤15% were imputed by single imputation. Subject matching was performed using the method of matching to the nearest available propensity score. That is, patients in the PCI population were randomly ordered and the first patient from PCI was then matched to a patient in the medical population with the nearest available Mahalanobis distance. The matched pair was then removed from the pool. The procedure was repeated until all patients in the PCI cohort were matched in a 1:1 fashion similar to prior methods.^12^ Performance of the propensity matching methods was assessed by estimating the absolute standardized differences across model covariates before and after propensity matching with a difference of <10% indicating clinically insignificant residual bias. The cumulative rate of outcomes was estimated in the propensity‐matched cohort using the Kaplan–Meier (KM) method and compared between groups using the log‐rank test. The mortality hazard ratio (HR) and 95% confidence interval (CI) for PCI versus medical therapy alone were estimated using Cox regression analyses. For all comparisons, *P*‐values <0.05 were considered statistically significant. Data were analysed using SAS version 9.2 (SAS Institute, Cary, NC).

To assess outcomes for patients receiving PCI in the contemporary era, we performed exploratory analyses that compared patients receiving drug‐eluting stents (instead of balloon angioplasty or bare metal stents) with those receiving medical therapy.

## Results

**Figure 1:**
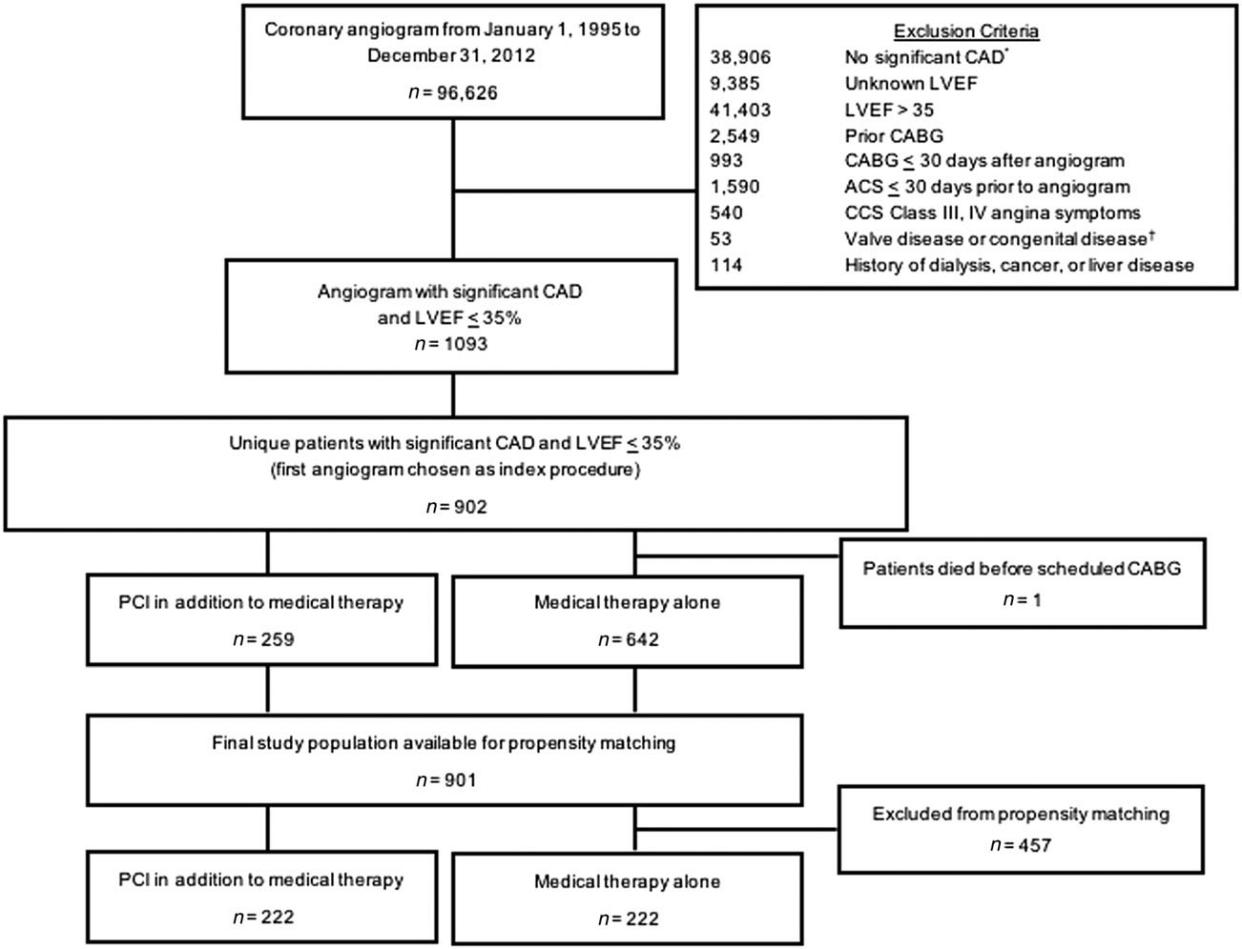
Flow diagram of the study design. This figure displays the initial study population, through exclusions, to the final study population. ^*^Prior to 1 July 2007, significant coronary stenosis was considered ≥75%. After this date, significant was considered ≥50%. ^†^We excluded patients with valve disease or congenital heart disease as the primary indications for cardiac catheterization. ACS, acute coronary syndrome; CABG, coronary artery bypass grafting; CAD, coronary artery disease; CCS, Canadian Cardiovascular Society angina classification system; LVEF, left ventricular ejection fraction; PCI, percutaneous coronary intervention.

There were 96 626 coronary angiograms performed at Duke University Medical Center from 1 January 1995 to 31 December 2012. Of these, there were 901 unique patients with stable CAD potentially amenable to PCI in ≥1 vessel and LVEF ≤35% without other exclusion criteria available for propensity score matching (*Figure* 1).

Patient characteristics of the full study population (i.e. prior to propensity score matching) stratified by treatment strategy are shown in *Table* 1. Of 901 patients, 259 (29%) were treated with PCI and 642 (71%) were treated with medical therapy. Patients treated with medical therapy alone had markers of more complex coronary disease including a longer history of CAD, more 3‐vessel disease, and more left main disease. Patients treated with medical therapy alone also had high‐risk features of HF including lower LVEF, worse New York Heart Association symptom class, and more grade 3 and 4 mitral valve regurgitation. Patients treated with medical therapy alone were also less likely to be treated with appropriate medical therapy, including aspirin or clopidogrel, angiotensin‐converting enzyme inhibitors or angiotensin II receptor blockers, beta‐blockers, or statins.

**Table 1:** Baseline characteristics of patients prior to propensity matching

	All patients (*n* = 901)	Medical therapy (*n* = 642)	PCI (*n* = 259)	*P*‐value
Age (years)	64 (55, 73)	64 (55, 73)	63 (53, 73)	0.32
Male sex	72	72	71	0.86
White race	64	62	71	0.01
Body mass index (kg/m^2^)	28 (24, 32)	27 (24, 31)	28 (24, 32)	0.05
CAD history (months)	49 (4, 127)	61 (7, 144)	20 (1, 89)	<0.01
Number of vessels^a^				<0.01
1	46	43	51	
2	28	27	33	
3	26	30	15	
Left main disease	10	13	5.0	<0.01
LVEF (%)	26 (20, 31)	25 (20 31)	29 (25, 32)	<0.01
Prior history of MI	34	32	39	0.02
NYHA class at angiogram				<0.01
I	6.5	6.9	5.5	
II	13	14	9.8	
III	25	28	18	
IV	19	23	11	
Hypertension	69	69	68	0.76
Diabetes	39	38	42	0.25
CVD	12	13	8.9	0.06
PAD	11	12	9.3	0.20
Any history of smoking	58	58	58	0.92
Charlson index^b^ >0	57	57	56	0.78
MV regurgitation^c^				0.01
1+	22	21	26	
2+	22	24	18	
3–4+	17	19	11	
Exam prior to angiogram				
SBP (mmHg)	135 (120, 153)	135 (118, 153)	137 (121, 155)	0.33
Carotid bruit	6.5	6.7	5.8	0.62
S_3_ or S_4_ on auscultation	16	18	11	0.01
Medications				
Aspirin or clopidogrel	87	83	99	<0.01
ACE‐I or ARB	77	73	87	<0.01
Beta‐blocker	76	69	91	<0.01
Spironolactone	22	23	20	0.36
Any diuretic	74	75	70	0.10
Hydralazine	9.3	8.7	11	0.33
Nitrates	37	35	43	0.03
Statin	57	50	76	<0.01
Warfarin	19	21	14	0.02

Values shown are medians (25th, 75th percentile) or percentages. ACE‐I, angiotensin‐converting enzyme inhibitor; ARB, angiotensin II receptor blocker; CAD, coronary artery disease; CVD, cerebrovascular disease; Exam, examination; LVEF, left ventricular ejection fraction; MI, myocardial infarction; MV, mitral valve; NYHA, New York Heart Association; PAD, peripheral arterial disease; PCI, percutaneous coronary intervention; SBP, systolic blood pressure.

a Prior to 1 July 2007, significant coronary stenosis was considered ≥75%. After this date, significant was considered ≥50%.

b Modified from the original Charlson index^13^, ^14^ of co‐morbidities to exclude cardiovascular components.

c Grade of mitral valve regurgitation determined by either ventriculogram or echocardiogram.

The absolute standardized differences across model covariates before and after propensity matching are displayed in the Supporting Information, *Figure* *S1*. After propensity score matching, none of the model covariates had a standardized difference > 10%. Patient characteristics of the propensity score‐matched population stratified by treatment strategy are shown in *Table* 1. This table also includes year of diagnostic angiogram, which was included in the propensity score model. As expected, baseline demographic characteristics, clinical characteristics including details of ventricular function and coronary anatomy, and medication use were well balanced between the two groups. Only one variable, duration of CAD, remained significant after propensity score matching (*Table* 2).

**Table 2:** Baseline characteristics of patients after propensity matching stratified by medical therapy or percutaneous coronary intervention

	Medical therapy (*n* = 222)	PCI (*n* = 222)	*P*‐value
Age (years)	64 (55, 73)	63 (54, 73)	0.91
Male sex	74	71	0.46
White race	69	69	1.00
Body mass index (kg/m^2^)	28 (24, 32)	28 (24, 32)	0.54
Duration of CAD (months)	61 (7, 144)	17 (1, 89)	<0.01
No. of vessels with CAD^a^			0.58
1	53	51	
2	33	32	
3	14	18	
Left main disease	8.1	5.4	0.26
LVEF (%)	28 (22, 32)	28 (23, 32)	0.60
Prior history of MI	38	36	0.69
NYHA class at angiogram			0.93
I	5.0	6.4	
II	9.9	11	
III	21	21	
IV	15	13	
Hypertension	68	70	0.61
Diabetes	41	43	0.63
Cerebrovascular disease	11	10	0.76
Peripheral arterial disease	10	11	0.88
Any history of smoking	60	56	0.44
Modified Charlson index^b^ >0	56	58	0.63
Mitral valve regurgitation^c^			0.93
1+	26	24	
2+	22	20	
3–4+	9.7	10	
Physical exam prior to angiogram			
SBP (mmHg)	136 (119, 152)	137 (121, 152)	0.58
Carotid bruit	8.1	6.4	0.47
S_3_ or S_4_ on auscultation	11	12	0.77
Medications			
Aspirin or clopidogrel	99	99	1.00
ACE‐I or ARB	85	86	0.79
Beta‐blocker	87	89	0.56
Spironolactone	23	20	0.49
Any diuretic	78	71	0.06
Hydralazine	8.6	11	0.42
Nitrates	41	42	0.85
Statin	73	73	1.00
Warfarin	23	15	0.03
Year of diagnostic angiogram			0.88
1995	3.6	4.5	
1996	4.5	4.5	
1997	6.3	5.9	
1998	8.1	7.2	
1999	5.9	7.7	
2000	5.9	7.7	
2001	5.0	5.0	
2002	4.5	6.3	
2003	8.1	7.7	
2004	5.4	6.3	
2005	4.1	4.5	
2006	5.4	5.0	
2007	7.2	4.1	
2008	6.8	5.0	
2009	4.5	4.5	
2010	4.5	4.1	
2011	6.8	3.2	
2012	3.6	7.2	

Values shown are medians (25th, 75th percentile) or percentages. ACE‐I, angiotensin‐converting enzyme inhibitor; ARB, angiotensin II receptor blocker; CAD, coronary artery disease; Exam, examination; LVEF, left ventricular ejection fraction; MI, myocardial infarction; NYHA, New York Heart Association; PCI, percutaneous coronary intervention; SBP, systolic blood pressure.

a Prior to 1 July 2007, significant coronary stenosis was considered ≥75%. After this date, significant was considered ≥50%.

b Modified from the original Charlson index^13^, ^14^ of co‐morbidities to exclude cardiovascular components.

c Grade of mitral valve regurgitation determined by either ventriculogram or echocardiogram.

**Figure 2:**
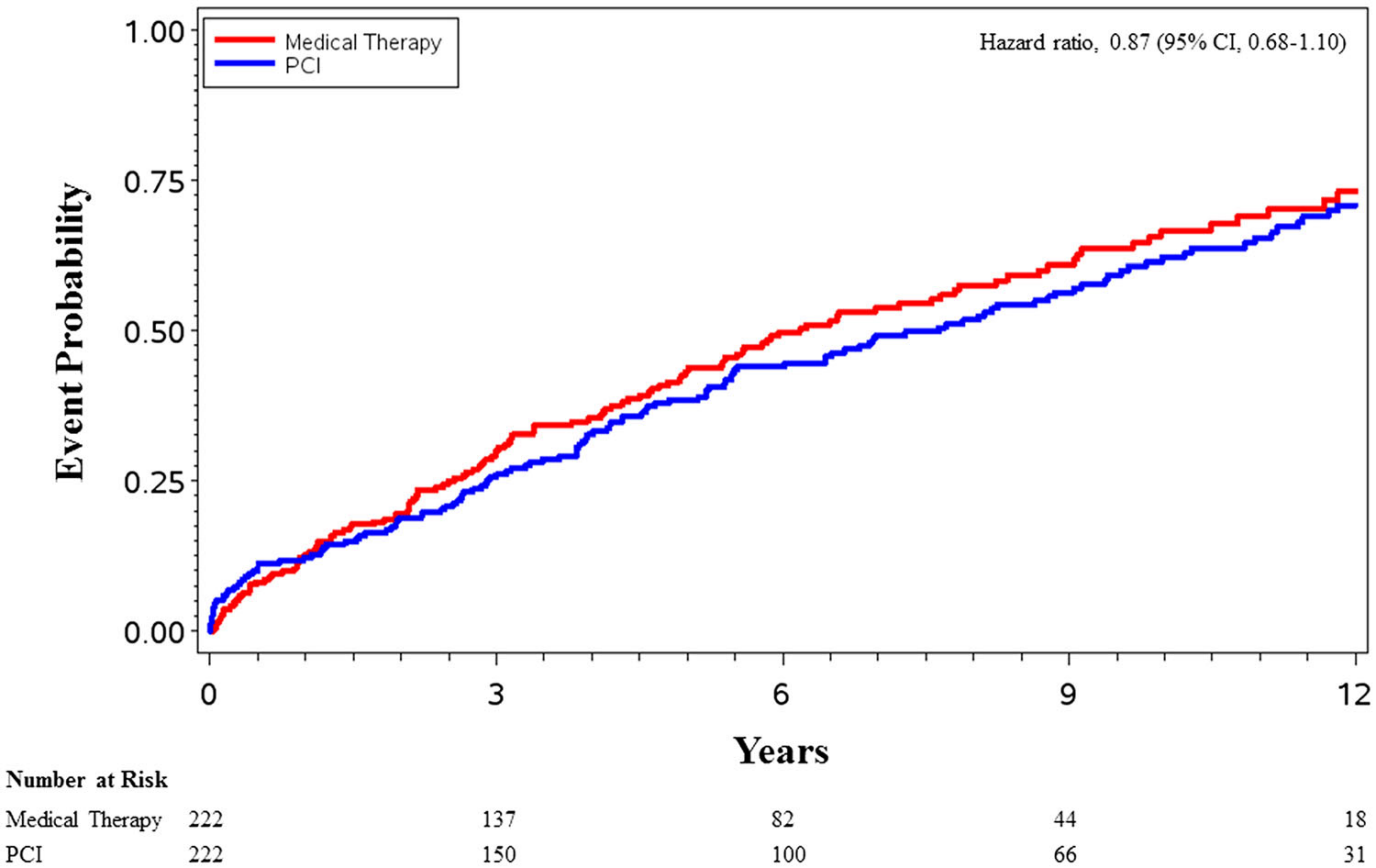
Probability of all‐cause mortality. Kaplan–Meier curves for the probability of all‐cause mortality. CI, confidence interval; PCI, percutaneous coronary intervention.

**Figure 3:**
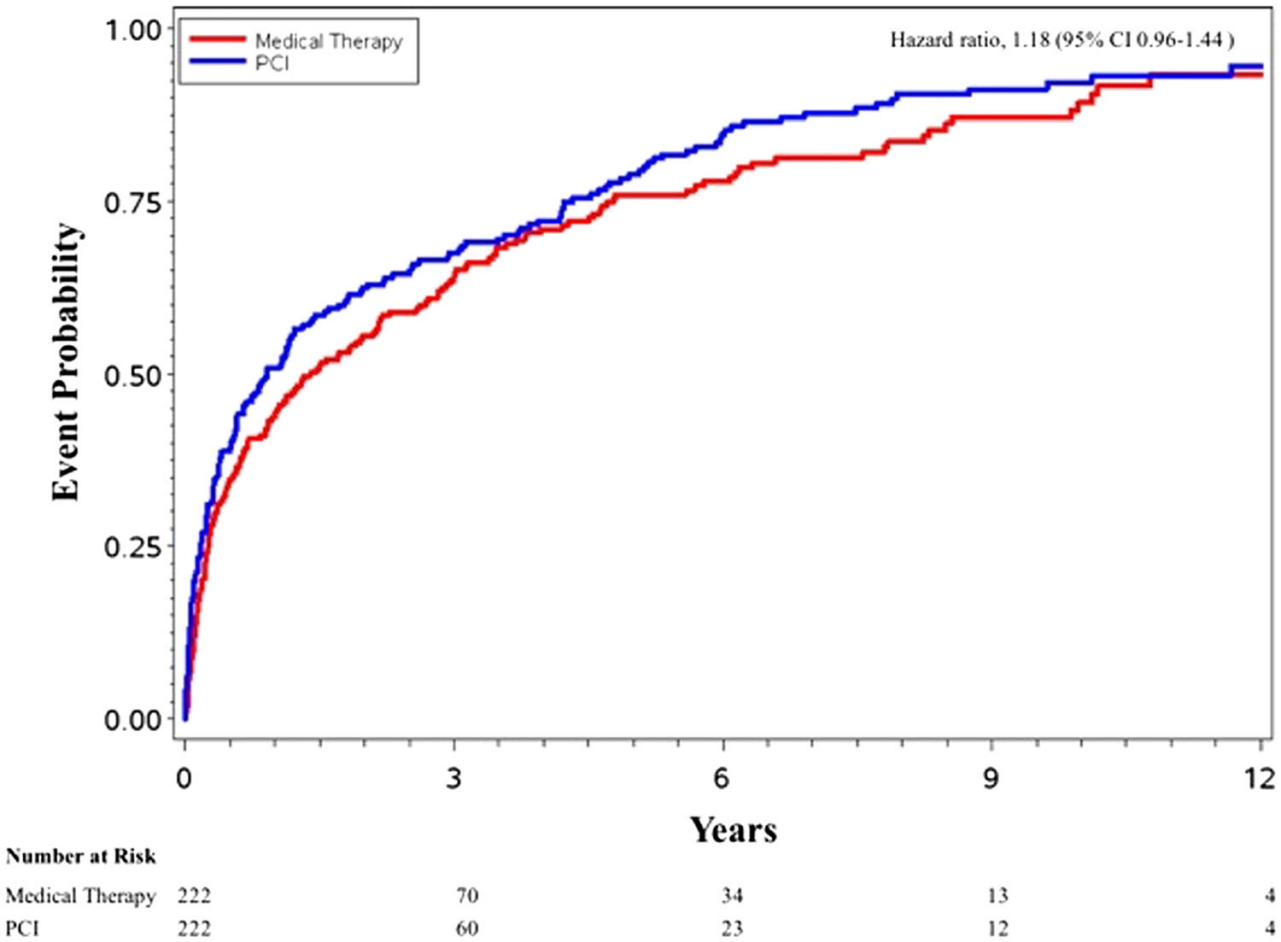
Probability of all‐cause mortality or cardiovascular hospitalization. Kaplan–Meier curves for the probability of all‐cause mortality or cardiovascular hospitalization. CI, confidence interval; PCI, percutaneous coronary intervention.

In the propensity‐matched cohort, the median follow‐up was 7.0 years (25th, 75th percentile 3.2, 11.1). The primary event of interest, all‐cause mortality, occurred in 27 patients (KM rate 0.12; 95% CI 0.09, 0.15) treated with PCI compared with 28 patients (KM rate 0.13; 95% CI 0.09, 0.18) treated with medical therapy at 1 year and occurred in 128 patients (KM rate 0.71; 95% CI 0.63, 0.78) treated with PCI compared with 125 patients (KM rate 0.73; 95% CI 0.65, 0.81) treated with medical therapy at 12 years (*Figure* 2); the HR was 0.87 (95% CI 0.68, 1.10). The composite endpoint of all‐cause mortality or cardiovascular hospitalization occurred in 119 patients (KM rate 0.54; 95% CI 0.47, 0.60) treated with PCI compared with 98 patients (KM rate 0.44; 95% CI 0.38, 0.51) treated with medical therapy at 1 year and occurred in 194 patients (KM rate 0.96; 95% CI 0.91, 0.98) treated with PCI compared with 181 patients (KM rate 0.93; 95% CI 0.87, 0.97) treated with medical therapy at 12 years (*Figure* 3); the HR was 1.18 (95% CI 0.96, 1.44).

In an exploratory analysis, we further evaluated patients that received drug‐eluting stents. Of the 222 patients treated with PCI, 58 (26%) received a drug‐eluting stent, 135 (61%) received a bare metal stent, and 29 (13%) received balloon angioplasty alone. To assess outcomes for patients receiving a PCI with contemporary interventional techniques, we performed an analysis that compared patients receiving drug‐eluting stents (instead of bare metal stents or balloon angioplasty) with those receiving medical therapy. In this subset of the overall population, patients treated with PCI in addition to medical therapy, as compared with medical therapy alone, had a similar rate of all‐cause mortality [HR 0.78 (95% CI 0.51, 1.20)].

## Discussion

The role of revascularization with PCI in addition to medical therapy as a treatment strategy for patients with LVSD in the absence of acute coronary syndrome and among those not treated with CABG remains unclear.^8^ In this observational study, patients treated with PCI in addition to medical therapy, as compared with medical therapy alone, had less complex coronary disease, markers of less HF risk, and were more likely to be treated with appropriate medical therapy for LVSD. Despite this finding, after propensity score matching, there was no significant difference between medical therapy alone and medical therapy plus PCI with respect to long‐term mortality and rehospitalization. Other possible differences in symptoms, functional status, or dependence on HF medications could not be documented based on available data. While PCI did not demonstrate any harm, these results challenge the value of PCI as a treatment strategy for patients with moderate or severe LVSD. Notably, the time frame of this report includes mostly patients treated in the era of bare metal and plain balloon PCI (74% of the PCI cohort), prior to the era of routine dual antiplatelet therapy after PCI. Our findings justify an adequately powered randomized clinical trial to further evaluate PCI as a treatment strategy in the contemporary PCI era with drug‐eluting stents and adjunctive antiplatelet therapy to assess the impact of PCI on long‐term mortality and cardiovascular hospitalization events, as well as other patient‐centred outcomes such as symptoms and functional status.

Despite being a common clinical issue, additional data comparing PCI with medical therapy for patients with LVSD are lacking. A meta‐analysis comparing revascularization strategies in addition to medical therapy with medical therapy alone in patients with LVSD found that compared with medical therapy alone, there was a significant mortality reduction with CABG [HR 0.66 (95% CI 0.61, 0.72); *P* < 0.001] and PCI [HR 0.73 (95% CI 0.62, 0.85); *P* < 0.001].^15^ Notably, the included data on PCI compared with medical therapy alone are from the two studies: Heart Failure Revascularization Trial (HEART)^16^ and a study of health system data published in abstract form.^17^ The HEART trial randomized patients with LVEF ≤35% and viable myocardium to medical management or angiography with intent to revascularize by either CABG or PCI.^16^ Due to slow enrolment, this study only randomized 138 of the planned 800 prior to early termination.

Additional data are available from the Alberta Provincial Project for Outcomes Assessment in Coronary Heart Disease registry.^18^ Patients with clinical HF (25% of which had a documented LVEF <35%) and CAD that underwent any revascularization (60% by CABG and 40% by PCI) were compared with medical therapy alone, and patients treated with PCI had better survival [adjusted HR 0.58 (95% CI 0.49, 0.69)] than those treated with medical therapy alone. Notably, acute coronary syndrome patients were included in the analysis. Our study was intentionally limited to patients without acute coronary syndrome or CCS class III or IV angina.

Outside of a randomized clinical trial context, the effect of PCI in addition to medical therapy cannot be established. Fortunately, additional evidence will be provided by the Study of Efficacy and Safety of PCI to Improve Survival in HF (REVIVED‐BCIS2), currently enrolling in the United Kingdom.^19^ In REVIVED‐BCIS2, investigators are randomizing patients with LVEF ≤30%, CAD potentially amenable to PCI, and viable myocardium (as assessed by dobutamine stress echocardiography or magnetic resonance imaging) to PCI in addition to medical therapy or medical therapy alone. A better understanding of the role of revascularization in addition to medical therapy in the management of HF patients with ischaemic myopathies may have important implications for patient outcomes, particularly because a prior analysis found that 73% of patients with new‐onset HF did not receive an evaluation for CAD within 90 days of the index admission for HF.^20^

Our study results are contrary to our original hypothesis that was based on prior data supporting revascularization as a treatment strategy for patients with LVSD. The STICH trial evaluated CABG in patients with CAD and LVSD. STICH demonstrated that the addition of CABG to optimal medical therapy did not affect 5‐year mortality rates compared with medical therapy alone, but did improve rates of cardiovascular death, as well as a composite of all‐cause mortality or cardiovascular hospitalization.^6^ In addition, patients with more advanced forms of ischaemic cardiomyopathy (i.e. 3‐vessel disease, more severe LVSD, and remodelling) derived the greatest benefit from CABG compared with medical therapy alone.^21^ STICHES was the long‐term follow‐up study to STICH, which found that the rate of all‐cause mortality over 10 years was lower among patients who underwent CABG in addition to receiving medical therapy than among those who received medical therapy alone [HR 0.79 (95% CI 0.66, 0.93)].^7^ In STICHES, CABG was associated with an incremental median survival benefit of nearly 18 months and prevention of one all‐cause death for every 14 patients treated. PCI has less upfront risk than CABG, and previous analyses suggest similar outcomes for PCI and CABG in patients with LVSD.^22^, ^23^ Significantly, STICH patients were enrolled prospectively in a multicentre, randomized, highly structured clinical trial, while our study retrospectively examined a longitudinal registry in which patients who underwent CABG were not included; therefore, the role of PCI in patients with moderate or severe LVSD is not completely addressed by our data.

One possible reason why PCI did not improve long‐term outcomes in our study is the inclusion of patients prior to the widespread use of drug‐eluting stents (we included patients treated from 1995 to 2012). The Food and Drug Administration first approved drug‐eluting stents in 2002, and their use rapidly increased to 90% of all coronary stent procedures in the United States by 2005, although these rates fluctuated over time due to safety concerns.^24^ Outcomes may be better with contemporary interventional techniques including drug‐eluting stents and intravascular ultrasound, as well as a deeper understanding of dual antiplatelet therapy's impact and the development of safer antiplatelet agents. Alternatively, the benefits seen in STICH may not be translatable to revascularization by PCI; however, the exclusion of CABG‐eligible patients from this report leaves the role of PCI as an alternative to CABG among patients with LVSD as an open question for additional study.

Our study must be considered in light of some limitations. First, this was a retrospective, observational analysis of single centre data; despite propensity score adjustment, other measured and unmeasured variables may have influenced study results. Second, during the study period, there were marked improvements in PCI technology for CAD and medical therapy for LVSD, although we did include the year of diagnostic angiogram in our propensity score model. Third, our definition of significant stenosis changed during the course of the study and is not the same as current guidelines.^25^ Fourth, we were unable to assess for all periprocedural complications including contrast‐induced acute nephropathy. Fifth, due to our study's sample size and the high event rate in our population, there was a small cohort of patients with data >9 years, limiting our ability to draw conclusions on outcomes to this duration. Finally, an important goal of revascularization is improvement in health status and quality of life, and these data were not available.^26^

In conclusion, in this well‐profiled, propensity‐matched cohort of patients with stable CAD potentially amenable to PCI and LVSD, there were no differences in long‐term mortality or the composite of all‐cause mortality or cardiovascular hospitalization between patients treated with medical therapy alone compared with medical therapy plus PCI. Nonetheless, a minority of patients revascularized with PCI were treated with contemporary interventional techniques (including drug‐eluting stents), and CABG‐eligible patients (such as those enrolled in STICH) were excluded from this analytic cohort. In the absence of any concerning safety signal, more informative prospective studies are needed to understand the benefit of PCI in this high‐risk patient population.

## Conflict of interest

The following relationships exist related to this manuscript: Dr. DeVore reports the following disclosures: Research: American Heart Association, Amgen, and Novartis; consulting: Novartis. Dr. *E. Magnus* Ohman reports the following disclosures: Research: Daiichi Sankyo (significant), Eli Lilly and Company (significant), and Gilead Sciences (significant); consulting: Abiomed (significant), AstraZeneca (modest), Daiichi Sankyo (modest), Eli Lilly and Company (modest), Gilead Sciences (modest), Janssen Pharmaceuticals (significant), Pozen, Inc. (modest), Sanofi Aventis (significant), The Medicines Company (modest), and WebMD (significant). A continuously updated list of disclosure information for Dr. Ohman is available at https://www.dcri.org/about-us/conflict-of-interest. Dr. Eric Velazquez reports the following disclosures: Research: Ikaria Pharmaceuticals (significant); consulting: Novartis Pharmaceuticals (significant).

## Funding

This study was sponsored internally by the Duke Clinical Research Institute, Durham, NC, USA.

## Supporting information


**Figure S1.** Standardized difference before and after propensity score matching
This figure displays the standardized difference for variables included in the propensity model before (blue) and after (red) propensity score matching.ACE‐I, angiotensin‐converting enzyme inhibitor; ARB, angiotensin receptor blocker; ASA, aspirin; BMI, body mass index; Cath, catheterization; Clop, clopidogrel; CVD, cardiovascular disease; LVEF, left ventricular ejection fraction; MI, myocardial infarction; Modified Charlson, modified from the original Charlson index^12,13^ of comorbidities to exclude cardiovascular components; MV, mitral valve; NYHA, New York Heart Association Functional Classification; PAD, peripheral arterial disease.
