# Supplementary figures and images for: Percutaneous coronary intervention outcomes in patients with stable coronary disease and left ventricular systolic dysfunction
Source: ESC Heart Fail. 2019 Sep 27;6(6):1233–42. doi: 10.1002/ehf2.12510 (PMC6989282; doi:10.1002/ehf2.12510)

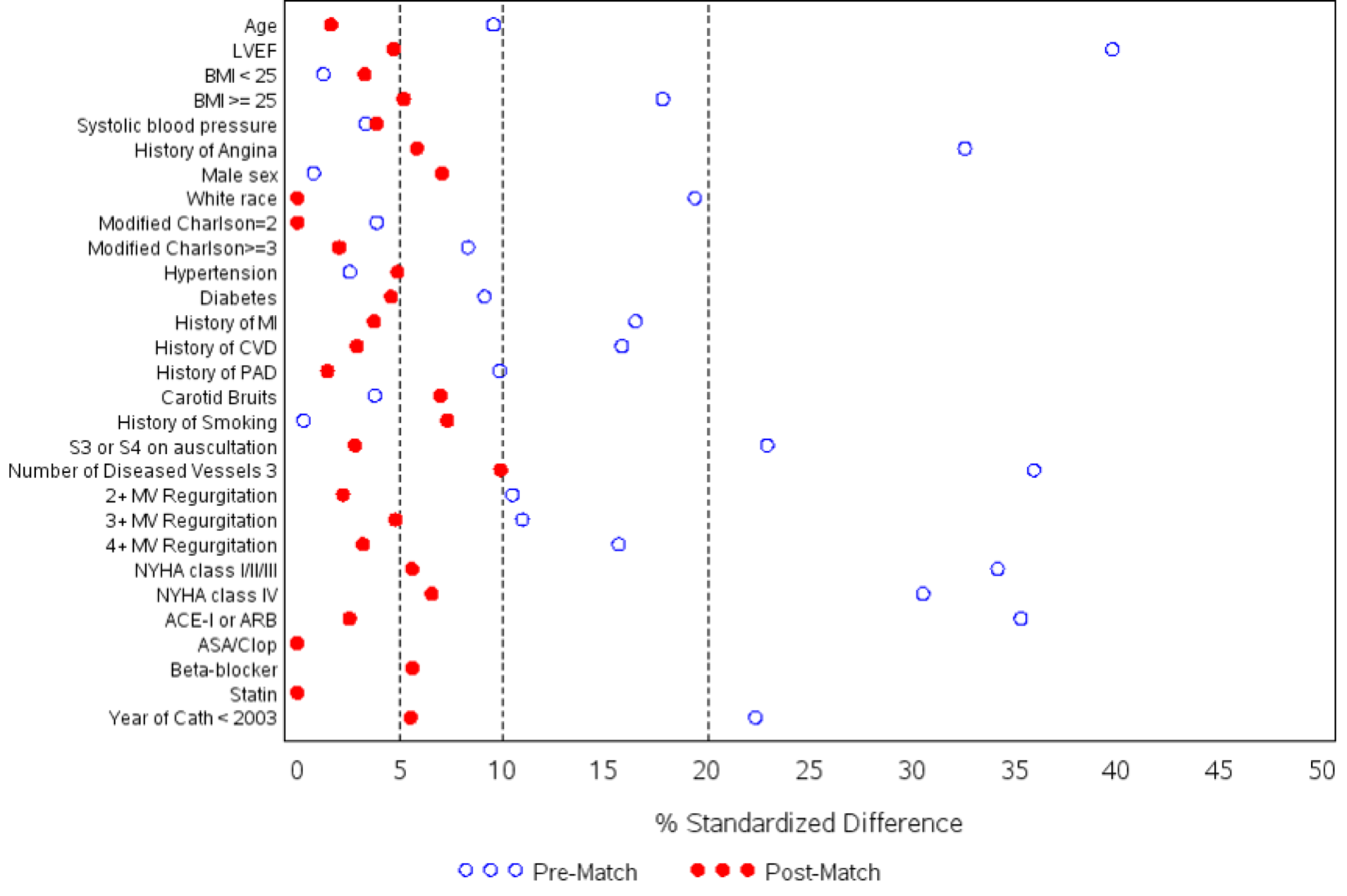

Supplement: Supplementary file 1 — Figure S1. Standardized difference before and after propensity score matching This figure displays the standardized difference for variables included in the propensity model before (blue) and after (red) propensity score matching.ACE‐I, angiotensin‐converting enzyme inhibitor; ARB, angiotensin receptor blocker; ASA, aspirin; BMI, body mass index; Cath, catheterization; Clop, clopidogrel; CVD, cardiovascular disease; LVEF, left ventricular ejection fraction; MI, myocardial infarction; Modified Charlson, modified from the original Charlson index12,13 of comorbidities to exclude cardiovascular components; MV, mitral valve; NYHA, New York Heart Association Functional Classification; PAD, peripheral arterial disease. [file EHF2-6-1233-s001.tiff]
